# Editorial: Diagnosis, prevention and treatment in diabetic nephropathy, volume II

**DOI:** 10.3389/fendo.2023.1142285

**Published:** 2023-01-19

**Authors:** Andrea Leonardo Cecchini, Federico Biscetti, Maria Margherita Rando, Andrea Flex

**Affiliations:** Cardiovascular Internal Medicine Unit, Fondazione Policlinico Universitario A. Gemelli IRCCS, Roma, Italy

**Keywords:** diabetes mellitus, diabetic nephropathy, chronic kidney disease, end stage kidney disease (ESKD), kidney

The number of diabetic patients worldwide has more than tripled in the past two decades, and approximately one-third of people with diabetes mellitus (DM) eventually develop diabetic kidney disease (DKD), of which approximately 50% may progress to end-stage disease (ESRD). DKD is the most relevant microvascular complication of diabetes together with retinopathy and neuropathy. ESRD is associated with a high cardiovascular mortality rate, risk of hospitalization, and all-cause mortality in patients with diabetes and places a huge economic burden on patients and society. In addition, ESRD harms patients’ psychological status due to disabling morbidity and a large amount of disability-adjusted life years (DALYs).

While the global spread of DKD and its consequences are certain, a full understanding of the pathophysiology, diagnosis, and treatment of this condition is still lacking.

So far, a combination of inflammation and insulin resistance was thought to be responsible for the development of DKD and its cardiovascular (CV) complications, including death. Furthermore, clinical assessment and traditional biomarkers (such as eGFR and proteinuria) have been considered convenient tools to suspect DKD and monitor CKD progression. While pathological sample analysis has remained the gold standard method for diagnosis. The introduction of a new panel of biomarkers into clinical practice has been hampered by the resulting poor accuracy and specificity achieved until now. Finally, comprehensive management of DKD-promoting risk factors has been considered the cornerstone recommendation of current CKD treatment guidelines in DM.

However, recently an increasing body of evidence is showing critical gaps and open questions about this interesting Research Topic, and these new findings could change our current certainties, particularly regarding the diagnosis and treatment of DKD. Nearly 50% of patients with type 2 DM in stage 3 chronic kidney disease (CKD) remain undiagnosed. Traditional biomarkers, considered solid tools for diagnosis, are lacking in terms of sensitivity and specificity and the limitations of their therapeutic and prognostic value significantly condition their role in clinical practice. Additionally, the renal puncture is not widely available due to its invasiveness. Furthermore, even when DKD is diagnosed, there is still no effective therapy, and the only treatment options for late-stage DKD include dialysis or kidney transplantation, which are expensive and significantly increase personal and social burdens. Moreover, DKD displays such clinical heterogeneity that it increases the difficulty of treatment, and even considerable effort to control risk factors and manage blood glucose has limited efficacy in preventing DKD progression. Therefore, there is an urgent need for sensitive diagnostic tools to successfully identify people at risk for DKD, and effective preventive and therapeutic strategies to improve patient outcomes.

Fortunately, recent technological advancement and cost reductions for modern diagnostic tests can promisingly help clinicians obtain a timely diagnosis of DKD that can delay its progression to ESRD. Furthermore, new insights into the pathogenic mechanisms of DKD could provide more possible directions for new therapies for DKD.

A deep understanding of the pathogenic process behind the development of DKD could open up new disease-modifying therapies, and remarkable discoveries have recently emerged.

Prebiotics, probiotics, diet, and antibiotic use influence microbial composition. However, also DKD promotes dysbiosis, which has been shown to contribute to renal disease progression by activating intrarenal RAS, promoting inflammation and fibrosis through increased TMAO production, and worsening tubulointerstitial damage through regulation of cholesterol homeostasis. Han et al. were the first to comprehensively characterize the different bacterial compositions in DKD compared to non-DKD subjects. In particular, an enrichment of Hungatella and Escherichia genera and depletion of butyrate-producing bacteria was observed. This could be a new revealing pathogenic condition associated with developing DKD.

DKD promotes impairment of mechanisms regulating oxidative stress and Wu and Chen observed an increase in intracellular iron accumulation, glutathione depletion, and lipid peroxidation in patients with CKD and DM. This phenomenon is called ferroptosis and is defined as iron-dependent regulated cell death, which involves the regulation of genes and proteins.

UA is an independent, modifiable risk factor for chronic kidney disease and a cause of CV and non-CV complications in DKD. Huang et al. found that this “old new biomarker” can also accurately reflect abnormal glomerular and/or tubular function since approximately 90% of its excretion is due to the kidneys.

Lastly, lipid metabolism is known to cause CVD, but Lu et al. showed how new lipid biomarkers and lipid indices may be associated with an increased risk of DKD.

Diagnosis is an open challenge for DKD. An accurate estimation of the glomerular filtration rate is critical for diagnosis and is also crucial for the classification and management of patients with CKD. However, measuring glomerular filtration rate using clearance of inulin, technetium-99m-diethylene triamine pentaacetic acid, iohexol, or 125I-iothalamate is invasive, inconvenient, and expensive to use in daily practice.

Estimation of eGFR using equations (including those recommended in guidelines such as the CKD-EPIcr-cys equation) has limitations regarding accuracy in older adults and has shown significantly lower accuracy in individuals with diabetes than in the non-diabetic group. Specifically, Jiang et al. observed that CKD-EPIcr and CKD-EPIcys overestimated and underestimated GFR, respectively.

In recent years, technological progress has reduced the costs of highly innovative diagnostic techniques. For example, whole genome transcriptome analysis has been used extensively in the field of DKD and could prove crucial in identifying a simplified panel of serum biomarkers that can predict the risk of developing DKD. Indeed, Wang et al. achieved high diagnostic efficacy with the combination of two markers. The expression of REG1A and RUNX3 was significantly increased in blood samples from DKD patients and may be a novel predictor of renal disease in DM.

Furthermore, proteomics-based analysis facilitated the identification of novel target proteins suitable for DKD diagnosis and progression. In particular, Huang et. al through pressure circulation technology and pulseDIA proteomic analysis identified additional sensitive markers for early detection of kidney disease from the blood and urine of diabetic patients with CKD. Specifically, the authors found that autophagy-related protein NBR1 was significantly upregulated in early and advanced DKD, while ATG4B and VPS37A were significantly downregulated with the progression of DKD.

Also traditional well-known biomarkers have also shown a promising new role in diagnosis. Lu et al. found that apolipoprotein A1 (Apo A1), apolipoprotein B (Apo B), and lipid ratios may be associated with the onset of DKD to support diagnosis using readily available, noninvasive biomarkers.

At present, treatment options for DKD are limited and progression to ESRD appears inevitable. However, new findings on the pathogenesis of DKD could lead to promising new therapies. For instance, Wu and Chen noted that treating the processes underlying ferroptosis by modulating intracellular signaling pathways or using iron-chelating agents could be a new treatment option to slow the progression of DKD.

Lin et al. studied extracellular vesicles (EVs). They observed their beneficial effects on kidney injury stemming from their anti-apoptotic, anti-inflammatory, antioxidant, and anti-fibrotic role and their ability to modulate podocyte autophagy.

Furthermore, statins appear to exert some sort of beneficial effect on kidney damage by reducing proteinuria along with their ability to protect diabetic patients from cardiovascular events. However, they fail to delay the progression of DKD. Thus, novel therapies targeting novel lipid biomarkers could be a potent treatment for DKD(4).

Finally, promising new ways to monitor DKD progression and stratify individual risk can significantly change patient outcomes.

Shi et al. found that urinary IL-18 is associated with impaired carotid-femoral pulse wave velocity (cf-PWV), which is the current clinical gold standard measure of arterial stiffness and established cardiovascular risk marker in patients with T2D with DKD.

Interestingly, DKD affects body composition causing a gradual quantitative and qualitative deterioration of muscle mass. Therefore, sarcopenia is a common complication of DKD and worsens patient outcomes. Lin et al. observed a significant association between decreased rectus femoris cross-sectional area/increased visceral fat area and DKD progression, resulting in a promising and easily obtainable biomarker of prognosis.

Nutrition is confirmed to be one of the most relevant aspects to be investigated in our patients with chronic renal insufficiency. Indeed, as noted by Duan et al., T2DM patients with a reduced 25(OH) vitamin D level had worse renal function with an increased risk of DKD progression.

Finally, Huang et al. developed a new risk model that includes traditional risk factors that contribute to the early identification and prevention of complications in patients with DKD.

Overall, the published articles contribute to enriching current knowledge on the pathogenesis of DKD and the authors’ innovative findings could answer the open questions regarding the need for sensitive diagnostic tools and effective therapeutic strategies. It is hoped that this is a promising step towards significantly improving the prognosis and outcomes of patients with DKD.

## Author contributions

ALC, MMR, FB and AF wrote the article. FB and MMR participated as guest editors for manuscripts of the Research Topic, where they were not coauthors themselves. All authors listed have made a substantial, direct and intellectual contribution to the work, and approved it for publication.

